# Characteristics of Participants and Findings of the National Breast Cancer Early Detection Program in Saudi Arabia

**DOI:** 10.3390/cancers17213403

**Published:** 2025-10-22

**Authors:** Fatina Al Tahan, Shaker A. Alomary, Hani Tamim, Mohammad Alkaiyat, Haifa Nassri, Khaled Alkattan, Abdul Rahman Jazieh

**Affiliations:** 1 Cancer Control Program, Ministry of Health, Riyadh 12628, Saudi Arabia; tahanft@yahoo.com; 2 General Directorate of Health Programs and Chronic Diseases, Ministry of Health, Riyadh 11176, Saudi Arabia; shalomary@moh.gov.sa (S.A.A.); haifanassri@yahoo.com (H.N.); 3 College of Medicine, Alfaisal University, Riyadh 11533, Saudi Arabia; hani_t@hotmail.com (H.T.); kkattan@alfaisal.edu (K.A.); 4 Department of Internal Medicine, Clinical Research Institute, Biostatistics Unit, American University of Beirut Medical Center, Beirut 1107 2020, Lebanon; 5 Oncology Department, King Abdul Aziz Medical City, Riyadh 11426, Saudi Arabia; alkaiyatmo@gmail.com; 6 Cincinnati Cancer Advisors, Cincinnati, OH 45212, USA

**Keywords:** breast cancer, screening, characteristics, risk factors, mammography, mammograms, predictors, Saudi Arabia, BMI

## Abstract

**Introduction:** Breast cancer remains the most common cancer among women in Saudi Arabia and is often diagnosed at advanced stages due to delays in screening. This study aimed to examine the characteristics of women participating in a national breast cancer screening program, identify the predictors of positive mammography findings, and assess the potential association between body mass index (BMI) and breast cancer risk. **Methods:** Data were obtained from the Saudi National Breast Cancer Early Detection Program that ran from May 2013 to July 2016. The study included Saudi women aged 30 years and older who underwent screening mammography and clinical breast examination. Demographic, clinical, and reproductive data were collected, and multivariable logistic regression analysis was used to determine the predictors of a positive mammogram and breast cancer diagnosis. **Results:** A total of 8348 women were screened (median age: 50 years), with 60 cases of breast cancer detected (detection rate: 7.4 per 1000). Most participants were either self-referred (62.4%) or referred by health educators (30.9), and 29.5% were illiterate. Oral contraceptive use was more common among women with breast cancer compared to those without (78.3% vs. 64.2%, *p* = 0.02), and the duration of use was significantly longer (median: 84 vs. 60 months, *p* = 0.041). However, in multivariable analysis, only abnormal findings on clinical breast examination were significantly associated with a positive mammogram (OR 21.3, 95% CI 7.9–57.1, *p* < 0.0001), but no independent association was found between BMI and breast cancer. **Conclusions:** The national screening program demonstrated a strong detection rate, with clinical breast examination being the only independent predictor of a positive mammogram. Larger studies including more breast cancer patients with longer follow-ups are needed to further investigate the influence of BMI and hormonal factors on breast cancer risk in Saudi women.

## Simple Summary

Breast cancer is the most common cancer among women in Saudi Arabia and is often diagnosed at a late stage. This study analyzed data from over 8000 women who participated in a national breast cancer screening program between 2013 and 2016. Most participants were self-referred or referred by health educators, and nearly one-third were illiterate. Sixty breast cancer cases were detected, with a detection rate of 7.4 per 1000 screened women. Clinical breast examination was the only independent predictor of a positive mammogram result. Women with breast cancer were more likely to have used oral contraceptives and for longer durations, but body mass index (BMI) was not independently associated with breast cancer risk. These findings highlight the importance of clinical exams in breast cancer screening and suggest the need for larger studies to better understand the role of hormonal and lifestyle factors in breast cancer among Saudi women.

## 1. Introduction

Globally, around 2.1 million women develop breast cancer, and more than half a million women die from breast cancer each year, accounting for ~15% of all cancer-related deaths in women [1]. Screening methods such as breast self-examination (BSE), clinical breast examination (CBE), and radiological imaging of the breast using low-dose X-rays in mammography help detect breast cancer early [2], but delays in accessing healthcare lead to high morbidity and mortality in developing countries [3].

Delayed diagnoses lead to patients having more advanced stages of breast cancer with worse outcomes in developing countries compared to developed countries. While more than 70% of breast cancer patients in most high-income countries are diagnosed in stages I and II, only 20–50% of patients in the majority of low- and middle-income countries are diagnosed in these earlier stages [4,5,6]. To detect and improve outcomes of breast cancer or pre-cancer in asymptomatic women, it is imperative to understand the patient characteristics of those who accept screening invitations for breast cancer examinations.

According to the 2016 National Cancer Registry (NCR) statistics, in the Kingdom of Saudi Arabia (KSA), breast cancer was the most frequent cancer among women, accounting for 31.4% of all cancers, and the age-standardized rate (ASR) was 28.4 per 100,000 Saudi women [7]. Previously, KSA was believed to be a low-risk country for breast cancer, but recent studies have reported an increase in both the incidence and prevalence of breast cancer among younger age groups [7,8].

This represents a significant disparity compared with developed countries. Five-year survival rates are still very low in countries with limited resources and where cancer is diagnosed at later stages; this is in contrast to countries where early detection and treatment are easily accessible, in which the five-year survival rate in early-stage cancers exceeds 80% compared to 10–40% in advanced stages [9]. Every woman has the right to be educated and aware of early detection and screening methodologies for breast cancer, but these strategies must be culturally appropriate and population-specific. When resources become available, they should be invested in appropriate screening methods like mammography, as it reduces breast cancer mortality [2,6]. Declining breast cancer mortality rates—estimated to be around 25.8% to 40% in North America and Taiwan—can be attributed to successful mammographic screening programs [10,11,12]. Women aged 50–69 years who use mammography screening services have, on average, about a 40% reduced risk of mortality from breast cancer [2]. In Saudi Arabia, mammography has been successfully utilized for the detection of breast cancer since 2002 [13]. In a survey of 10,735 in 2013, 1135 were women aged 50 years or older, and about 89% of women did not have a clinical breast exam in the past year, and 92% never had a mammogram [14]. However, studies showed that there has been a continuous rise in female breast cancer cases in KSA in the past years, with an 18.7% breast cancer-related mortality rate among Saudi women. As reported by the WHO, 2791 and 3629 BC cases were diagnosed in KSA in 2012 and 2018, respectively, and this number is predicted to reach 6886 cases in 2040 [15,16].

The breast cancer screening program was launched to assist in controlling breast cancer from awareness to treatment. Saudi statistics for the prevalence of breast cancer warned of a significant increase in the future, but most institutions focused on diagnosing, which usually occurs in advanced stages with significant morbidity and mortality. As a result, non-governmental pilot projects that included smaller numbers of patients were launched [17]. There was a national need to implement screening programs in different regions of the country, and support for these programs by Saudi women was encouraging.

This study evaluates the characteristics of women undergoing breast cancer screening through the National Breast Cancer Early Detection Program in Saudi Arabia. It aims to identify predictors of positive mammograms and assess the influence of various risk factors, thereby providing insights to enhance screening effectiveness and guide public health strategies.

## 2. Methodology

### 2.1. Study Setting

The study participants were selected from the ongoing national breast cancer screening program, “The National Breast Cancer Early Detection Program” (NBCS), which was established in 2012, with more than 61 clinics providing screening in 20 regions in Riyadh, Kingdom of Saudi Arabia. Two clinics were also situated in the 2 shopping centers in Riyadh. The program aimed to educate Saudi women about breast health, breast cancer awareness, and mammographic screening, thereby improving their overall health. Screening procedures and mammography were carried out in the clinics by well-trained medical professionals, including certified technicians with experience in mammograms and certified breast radiologists with reading rates for screening mammograms of no less than 3000/year.

### 2.2. Study Design

A survey of women participating in the ‘National Breast Cancer Early Detection Program’ was conducted between May 2012 and July 2016. The study population consisted of 8348 Saudi women aged 30 to 89 years. All asymptomatic women who were willing to participate in the National Breast Cancer Early Detection Program were recruited in the study as well as those who had one of the following risk factors: significant family history of a first-degree relative or two second-degree relatives with breast or ovarian cancer, genetic susceptibility, previous history of breast/ovarian cancer, or previous thoracic radiotherapy at during ages 10–30.

### 2.3. Data Collection Procedure and Tools

The National Scientific Committee of the program designed a comprehensive questionnaire that can be scored in terms of various demographic data, risk factors, lifestyles, family history, personal medical history, previous mammogram, clinical exam findings, and mammogram findings. A pilot test was conducted with a small group (20) of participants to evaluate the clarity, relevance, and value of the questions before finalizing them for use in a special digital software application called Breast Information System-Synapse v6.1, 2012, FujiFilm Corporation (Tokyo, Japan) (see Supplementary Materials).

Similarly to other clinical encounters, upon registration, women were interviewed by well-trained administrative personnel or health educators, and their responses were recorded electronically.

### 2.4. Anthropometric Measures

Height (centimeters on a fixed stadiometer), weight (kilograms), and body mass index (kg/m^2^) (BMI) were calculated using Quetelet’s index (weight in kilograms divided by height in meters squared) [18]. BMI (kg/m^2^) scores were classified according to WHO criteria, with the cut-off points being <18.50 kg (underweight), between 18.50 and 24.99 kg (normal weight), ≥25 kg (overweight), and ≥30.00 kg (obese) [19].

### 2.5. Clinical Data

#### 2.5.1. Clinical Breast Exam Findings

Clinical breast examinations were performed by qualified family physicians before the study screening/mammogram or immediately after the mammographic results were available. The clinical data included a change in breast size and shape, the presence of breast pain, nipple discharge, inversion, or pain, dimpled or scaly skin, changes in skin color, or lymph node enlargement. The presence of any of these symptoms was considered a positive exam and labeled as symptomatic. The primary care physicians or radiologists at the center performed the physical exam, scoring “Yes” for a positive exam and “No” for a negative exam. The clinical exam was completed by physicians who participated in the initial period of the program to enhance the credibility of the program; therefore, this was included as part of the screening program. Patients referred by their physicians for diagnostic mammograms were managed by the relevant healthcare systems and were not included in this program.

#### 2.5.2. Mammographic Findings

The Breast Imaging Reporting and Data System (BI-RADS) classification system indicates the level of suspicion for breast cancer [20,21]. Two breast radiologists independently interpreted the images (double-blind readings), and discrepancies were resolved by a consensus review. This system is known as an effective way to improve the quality of care by reducing errors or missed abnormalities and decreasing the recall rate.

The BI-RADS (Breast Imaging Reporting and Data System) categories for mammograms are as follows:

o 0—Incomplete, needs additional imaging;
o 1—Negative;
o 2—Benign finding;
o 3—Probably benign finding;
o 4—Suspicious abnormality;
o 5—Highly suggestive of malignancy;
o 6—Known biopsy-proven malignancy.

A positive mammogram indicates that there is a need for further workup and referrals to the diagnostic clinic, including mammograms reported using the BI-RADS system with scores correlating to categories 0, 4, and 5. A negative mammogram indicates normal or benign changes, with no additional work needed for BI-RADS categories 1 and 2. However, a cancer diagnosis is not made without a confirmed tissue diagnosis by breast lesion biopsy.

Information on reproductive history, lifestyle, and dietary and physical behaviors includes age at menarche and menopause, parity, number of children, history, duration of breastfeeding, use, duration of contraceptive pills, history of hormone replacement therapy, dietary pattern related to vegetables/fruits/meat, physical activity and sedentary behavior, certain dietary intake, and physical activities.

### 2.6. Study Population

The study population consists of women who underwent a screening mammogram in the Riyadh region of KSA. This national program is run by the Ministry of Health, which provides healthcare coverage for 70% of the population; therefore, there was continuous advertisement in the primary care centers, hospitals, and shopping malls, as well as multiple campaigns in collaboration with the Ministry of Education (in schools, especially high schools and universities) and Ministry of Information (via TV, radio, and social media). Permission and verbal informed consent were obtained before joining the screening program, and enrolled participants were interviewed after a thorough explanation of the study.

### 2.7. Data Management

Data from the information described above were coded and entered into datasets, which underwent data cleaning and duplication removal. A unique subject identifier was generated for each subject.

### 2.8. Statistical Analysis

Descriptive statistics were reported as either the mean and standard deviation (SD) for normally distributed continuous variables or the median and interquartile range (IQR) for skewed continuous variables, while frequency (%) was used for categorical variables. Descriptive statistics were analyzed by disease status for the entire group. Bivariate analysis was carried out to assess the association between different factors and disease status using the chi-squared test for categorical variables and the independent *t*-test for continuous variables. Logistic regression analysis was used to identify predictors of positive screening; variables that have been reported to be clinically or statistically significant were included in the model. Results are reported as odds ratios (ORs) with 95% confidence intervals (CIs). SPSS (Statistical Package for the Social Sciences) software v28 (SPSS, Chicago, IL, USA) was used for data cleaning, management, and analysis. *p*-values < 0.05 were considered to be statistically significant.

## 3. Results

### Participant Demographics

A total of 8348 participants were included in the analysis, of whom 60 patients were diagnosed with breast cancer, with a detection rate of 7.18 per 1000. The median age of the study participants was 50 (45–55) years, while the median BMI was 31.2 kg/m^2^ (24.9–35.8). A higher proportion of women were illiterate (29.5%), followed by participants with a college education (23.8), and those who finished primary (18.4%) and secondary (13.8%) school (Table 1). There was no significant difference between the groups without and with breast cancer. Most of the patients were self-referred or referred by health educators.

Various reproductive variables, as shown in Table 2, were recorded and analyzed. Only the use of birth control pills and their duration of use were significantly higher in the BC group.

Upon clinical examination, about 2.6% of women were positive for breast cancer. Upon breast examination, about 1.8% had a breast mass/lump. However, we did not observe any change in breast shape and/or breast pain in the majority of women who underwent screening. Similarly, the majority of the women had no nipple changes, such as nipple discharge, inversion, or pain. Moreover, upon skin/lymph node examination, the majority of women had no dimpled or scaly skin, changes in skin color, or lymph node enlargement (Table 3).

Over 90% of female participants were 40 years and older. The mammogram results showed that 42.2% of women had a normal mammogram with no lump, 52% had benign lumps, and 7.4% had suspicious lumps (Table 4). Among risk factors noted as the reason for the mammogram referrals, the majority of women (66.8%) had a family history of breast cancer in first-degree relatives, while 0.9% had a personal history of breast cancer and 0.2% had a family history of ovarian cancer. There was no difference between the groups in terms of having a history of breast biopsy or family history of breast and ovarian cancers in first- and second-degree relatives.

Except for the fat/oil intake, lifestyle factors such as fruit, vegetable, and meat intake did not differ between the two groups (Table 5).

Multivariable analysis revealed that physical exam results are the only significant factor for a positive mammogram finding (Table 6).

## 4. Discussion

Our real-world study included 8348 Saudi women who underwent screening mammography provided by a national screening program, of which 60 were diagnosed with breast cancer. Although the number of diagnosed cases is too small to accurately determine all the risk factors, the studies highlighted multiple important issues. The detection rate was 7.18 per 1000, which is higher than the average benchmark of 4.7 per 1000 [22]. This may be due to more cases being diagnosed during initial mammograms as a result of early screening in women with symptoms of breast cancer.

Participants have various levels of education, with the largest population of participants being illiterate, followed by college graduates. Studies conducted in Qatar, Nigeria, and Saudi Arabia suggest that an education level of high school or above was a significant predictor of knowledge and awareness of BCS [23,24,25,26]. Higher education levels were reported to be a predictor of undergoing mammography screening, but reaching women with lower education levels was a challenge for cancer screening [27,28]. Furthermore, when we explored the reasons for referrals for mammogram screening, age was one of the most important predictors, and a significant number of referrals were made by health centers/educators due to the potential limitations of self-examinations or clinical examinations [25]. The role of health educators allowed for reaching out to various sectors to invite 30% of participants, but the majority were self-referred, which highlights other effective public education and outreach efforts that were able to convince these women to seek screening [29]. Thus, healthcare centers play an important role in improving the cancer screening practices of women in Saudi Arabia.

Regarding the reproductive history and use of hormones, our study results indicate that most of the participants had menarche at an early age. A high proportion of participants used contraceptive pills, but the length of contraceptive use was not a significant risk factor because of the short duration of use overall. For women in menopause, the mean age was 51 years. An earlier study from Saudi Arabia assessing women’s knowledge regarding breast cancer reported that about 49.9%, 35.7%, and 33.1% knew that use of contraceptive pills, late menopause, and hereditary influences, respectively, were risk factors of breast cancer [23]. Therefore, the increased knowledge and awareness among women regarding the risk factors may have led them to undergo BCS.

Our study revealed that only the use of contraceptives and the duration of use were significant predictors for breast cancer diagnosis in our patient population, which is consistent with other findings in the literature [30,31,32].

For obvious reasons, abnormal findings on breast physical examination, such as the presence of a lump, changes in breast size or shape, nipple inversion, changes in skin color, or lymph node enlargement, were significant predictors of a positive mammogram and the only significant predictors in the multivariate analysis.

Age at menarche, menopause, and BMI were positively associated with breast cancer risk, and an inverse association was observed between age at menarche and breast cancer risk [33,34]. However, there are inconsistencies in published reports regarding the nature of the association between the age at menarche and mammographic findings, with some studies finding that increasing age at menarche contributed to higher breast density, whereas others reported no statistically significant association [33,35,36].

Multiple published studies have reported that the premenopausal state, nulliparity, a family history of breast cancer, and a history of HRT usage were all associated with positive mammographic results [18], but our study found that only the use of contraceptives was a predictor of positive mammograms.

As a part of this study, we aimed to assess the relationship between obesity and being overweight with breast cancer. Obesity is reported to be a risk factor for breast cancer and other cancers and is a very important issue to address in cancer education and prevention.

Multiple studies have evaluated the link between obesity and breast cancer at the molecular, enzymatic, and proteomic levels, including systemic studies [36]. A few studies have reported no association or a weak association of breast cancer with obesity in postmenopausal women [19,37,38,39,40]. On the other hand, recent studies showed a strong association, with one to two times the odds of breast cancer correlating with BMI (“A Matched Case–Control Study of Risk Factors for Breast Cancer Risk in Vietnam”) [41,42]. There are many reasons for these contrasting results. For example, BMI cut-offs have been used differently in various studies. Moreover, various methodological aspects such as study design, participant characteristics, definitions, screening techniques, and the heterogeneity of the baseline population could have affected the findings. Our study found a median BMI of 31.2 kg/m^2^ among participants, indicating a high prevalence of obesity within the screened population. This aligns with national data showing rising obesity rates among Saudi women, particularly those of lower socioeconomic status. Obesity is a recognized risk factor for postmenopausal breast cancer, primarily due to increased peripheral conversion of androgens to estrogens in adipose tissue, and global studies have reported that women with obesity have a 1.5- to 2-fold increased risk of developing postmenopausal breast cancer [43]. In the Saudi context, Elkum et al. (2014) found that 75.8% of breast cancer patients were overweight or obese compared to 61.3% of controls, supporting a significant association between BMI and breast cancer risk [44]. However, our study did not demonstrate a statistically significant association, possibly due to the relatively younger median age of our cohort or the limited number of cancer cases.

Family history of breast cancer was reported by 66.8% of participants, yet it did not emerge as a significant predictor of a positive mammogram. This contrasts with a comprehensive meta-analysis showing that a first-degree family history increases breast cancer risk by nearly twofold [45], which is a discrepancy that may reflect underreporting due to cultural perceptions or limited awareness of familial health histories. Additionally, the absence of genetic testing and counseling services limits the accurate identification of hereditary risk in this population. In Saudi Arabia, a recent study identified constitutional BRCA1 promoter methylation as being significantly associated with triple-negative breast cancer, underscoring potential genetic predisposition [46].

These findings highlight the importance of contextualizing breast cancer risk factors within local population dynamics. Future studies with larger cancer case samples, detailed pedigree data, and genetic testing integration could provide a more nuanced understanding of these associations in Saudi women.

Our study has several strengths and limitations. Firstly, this study was one of the first to be conducted in Saudi Arabia that assessed the characteristics of women undergoing breast cancer screening. Previous studies conducted in Saudi Arabia only evaluated the knowledge and awareness of BCS among women, but not the characteristics of women undergoing screening [23]. Secondly, we collected data from the National Breast Cancer Early Detection Program, which is an ongoing program in the Kingdom. Therefore, we have a representation of participants from different backgrounds, which makes our results generalizable to at least the Gulf countries. The main strength of our study is that it is a population-based study with an unselected sample, i.e., regardless of breast cancer status, covering a large sample size. This means that our results can be considered reliable and generalizable to a broader population since we gathered data covering most of the risk factors, confounding factors, and exposure of interest. These factors were also examined and included in the final analysis. Hence, these results are of great importance as most of the information was collected by trained health workers and doctors.

However, the limitations of our study include the nature of descriptive studies, as we were unable to test the relationships between the diagnosis of breast cancer and multiple risk factors. This may be due to the small sample size of patients who were diagnosed with breast cancer. However, descriptive studies can provide opportunities to generate hypotheses [47]. Another potential limitation is the retrospective collection of data, which could have led to the inability to recall information on certain important risk factors. Furthermore, another limitation is that self-reported data may potentially result in residual confounding. In addition, dietary intakes may have been insufficiently reported, especially because diet and breast cancer have been linked in several studies conducted in North America, Europe, and Asia [41,48,49,50,51].

Since our screening program included a clinical breast exam, patients with breast exam findings were included, but mammography data would have been included regardless. A clinical breast exam is not a typical component of a screening mammography program, but this finding highlights its importance.

## 5. Conclusions

Data concerning predictors of the positive outcomes of the mammogram in the Arab population are scarce. Through this large population-based study, we aimed to contribute to a further understanding of the literature, but due to the small number of patients diagnosed with cancer, it is difficult to generalize these findings without conducting a study with a larger sample size. Performing a clinical breast exam and providing counseling regarding the risk of contraceptives should be part of the standard of care.

### Recommendations

Since many of the patients were referred by healthcare educators/healthcare centers, we recommend increasing the prevalence of health education. Messages should be designed to tackle the fear of being diagnosed with breast cancer, and awareness of the importance of screening through clinical breast examination and mammography should be increased. Healthcare professionals must continue to remind and inform women of the risk factors of breast cancer in a culturally sensitive manner, and cancer screening practices must be upheld. Further longitudinal studies with large sample sizes and more study parameters are needed to understand the impact of various risk factors of breast cancer in Saudi Arabia.

## Figures and Tables

**Table 1:** Sociodemographic characteristics of study participants by breast cancer status. (N = 8348).

Variables	Total (N = 8348)	No Breast Cancer (N = 8288)	Breast Cancer (N = 60)	*p*-Value
Age (years) Median (IQR)	50.0 (45.0–55.0)	50.0 (45.0–55.0)	50.0 (46.0- 57.0)	0.49
**Body Mass Index (kg/m^2^)** *Median (IQR)*	31.2 (24.9–35.8)	31.2 (24.9–35.8)	32.5 (25.7–36.6)	0.40
**Educational Level**				0.11
Illiterate	974 (29.5)	964 (29.5)	10 (30.3)	
Primary	609 (18.4)	608 (18.6)	1 (3.0)	
Secondary	456 (13.8)	451 (13.8)	5 (15.2)	
High school	453 (13.7)	449 (13.7)	4 (12.1)	
College	788 (23.8)	775 (23.7)	13 (39.4)	
Post-graduate	25 (0.8)	25 (0.8)	0 (0.0)	
**Source of Referral**				
Self-referred	5208 (62.4)	5180 (62.5)	28 (46.7)	<0.0001
Doctor	64 (0.8)	60 (0.7)	4 (6.7)	
Family member	40 (0.5)	40 (0.5)	0 (0.0)	
Friend	28 (0.3)	28 (0.3)	0 (0.0)	
Health educator	2582 (30.9)	2560 (30.9)	22 (36.7)	
Call center	331 (4.0)	327 (3.9)	4 (6.7)	
Health center	45 (0.5)	44 (0.5)	1 (1.7)	
Other	50 (0.6)	49 (0.6)	1 (1.7)	

*p*-values were calculated using either the chi-square test or the independent *t*-test, where *p* < 0.05 is significant. IQR: interquartile range.

**Table 2:** Reproductive history of participants by breast cancer status (N = 8348).

Variables	Total (N = 8348)	No Breast Cancer (N = 8288)	Breast Cancer (N = 60)	*p*-Value
Age at menarche (years) *Median (IQR)*	13.0 (12.0–14.0)	13.0 (12.0–14.0)	13.0 (12.0–14.0)	0.21
Gone through menopause	3464 (41.5)	3437 (41.5)	27 (45.0)	0.58
Age at menopause (years) *Median (IQR)*	51.0 (47.0–54.0)	51.0 (47.0–54.0)	49.0 (48.0–52.0)	0.35
Marital status				0.19
Single	185 (2.2)	185 (2.2)	0 (0.0)	
Married	7371 (88.3)	7321 (88.4)	50 (84.7)	
Divorced	394 (4.7)	388 (4.7)	6 (10.2)	
Widowed	395 (4.7)	392 (4.7)	3 (5.1)	
Has been pregnant	7929 (95.0)	7870 (95.0)	59 (98.3)	0.37
Number of total pregnancies *Median (IQR)*	7 (5–10)	7 (5–10)	7 (5–10)	0.85
Age at first FTP/live birth *Median (IQR)*	20.0 (17.0–23.0)	20.0 (17.0–24.0)	21.0 (18.0–25.0)	0.474
Total number of FTPs *Median (IQR)*	7.0 (5.0–10.0)	7.0 (5.0–10.0)	6.0 (5.0–9.0)	0.551
Number of children *Median (IQR)*	7 (5–10)	7 (5–10)	6 (5–9)	0.808
History of breastfeeding	7149 (87.6)	7096 (87.6)	53 (88.3)	0.86
Length of lifetime breastfeeding (months) *Median (IQR)*	72.0 (30.0–156.0)	72.0 (30.0–156.0)	60.0 (24.0–120.0)	0.623
History of contraceptive pill use	5366 (64.3)	5319 (64.2)	47 (78.3)	**0.02**
Length of lifetime use of contraceptive pills *Median (IQR)*	60.0 (24.0–120.0)	60.0 (24.0–120.0)	84.0 (24.0–144.0)	**0.041**
History of hormone replacement therapy use	61 (0.7)	61 (0.7)	0 (0.0)	1.00

FTP: full-term pregnancy. *p*-values were calculated using either the chi-square test or the independent *t*-test, where *p* < 0.05 is significant. IQR: interquartile range.

**Table 3:** Clinical findings from clinical examinations among women undergoing breast cancer screening (N = 8348).

Variables	Total (N = 8348)	No Breast Cancer (N = 8288)	Breast Cancer (N = 60)	*p*-Value
Result of clinical examination				**<0.001**
Negative	8128 (97.4)	8086 (97.6)	42 (70.0)	
Positive	220 (2.6)	202 (2.4)	18 (30.0)	
Presence of breast mass/lump	150 (1.8)	133 (1.6)	17 (28.3)	**<0.001**
Change in breast size	3 (0.0)	1 (0.0)	2 (3.3)	**<0.001**
Change in breast shape	6 (0.1)	3 (0.0)	3 (5.0)	**<0.001**
Presence of breast pain	21 (0.3)	20 (0.2)	1 (1.7)	0.14
Presence of nipple discharge	30 (0.4)	30 (0.4)	0 (0.0)	1.00
Presence of nipple inversion	33 (0.4)	30 (0.4)	3 (5.0)	**<0.01**
Presence of nipple pain	3 (0.0)	3 (0.0)	0 (0.0)	1.00
Dimpled skin	1 (0.0)	1 (0.0)	0 (0.0)	1.00
Scaly skin	2 (0.0)	2 (0.0)	0 (0.0)	1.00
Changes in skin color	4 (0.0)	3 (0.0)	1 (1.7)	**0.03**
Lymph node enlargement	10 (0.1)	8 (0.1)	2 (3.3)	**<0.01**

*p*-values were calculated using the chi-square test, where *p* < 0.05 is significant.

**Table 4:** Mammogram findings among women undergoing breast cancer screening (N = 8348).

Variables	Total (N = 8348)	No Breast Cancer (N = 8288)	Breast Cancer (N = 60)	*p*-Value
Reason for mammogram referral				**<0.001**
Risk factor	161 (1.9)	160 (1.9)	1 (1.7)	
40 years old and above	7526 (90.2)	7487 (90.3)	39 (65.0)	
Symptomatic	52 (0.6)	50 (0.6)	2 (3.3)	
Risk factors + symptomatic	10 (0.1)	9 (0.1)	1 (1.7)	
Age + risk factors	488 (5.8)	482 (5.8)	6 (10.0)	
Age + symptomatic	111 (1.3)	100 (1.2)	11 (18.3)	
Risk factor reason for referral				0.31
One or more cases of breast cancer among first relatives	392 (66.8)	389 (67.1)	3 (42.9)	
Two or more cases of BC among second relatives	131 (22.3)	127 (21.9)	4 (57.1)	
History of breast biopsy	152 (1.8)	151 (1.8)	1 (1.7)	1.00
Mammogram screening result				**<0.001**
Negative (no finding)	3528 (42.4)	3525 (42.7)	3 (5.1)	
Negative (benign finding)	4177 (50.2)	4167 (50.4)	10 (16.9)	
Positive (suspicious finding)	618 (7.4)	572 (6.9)	46 (78.0)	

*p*-values were calculated using the chi-square test, where *p* < 0.05 is significant.

**Table 5:** Diet and physical activity of women undergoing breast cancer screening (N = 8348).

Variables	Total (N = 8348)	No Breast Cancer (N = 8288)	Breast Cancer (N = 60)	*p*-Value
Number of vegetable and fruit servings per day				0.65
None	158 (9.1)	155 (9.1)	3 (15.8)	
1 serving	850 (49.2)	840 (49.2)	10 (52.6)	
2–4 servings	654 (37.9)	648 (37.9)	6 (31.6)	
5 or more servings	65 (3.8)	65 (3.8)	0 (0.0)	
Number of vegetable and fruit servings per week				0.11
None	113 (5.8)	111 (5.8)	2 (10.0)	
1 serving	302 (15.5)	296 (15.3)	6 (30.0)	
2–4 servings	862 (44.2)	857 (44.4)	5 (25.0)	
5 or more servings	672 (34.5)	665 (34.5)	7 (35.0)	
Number of red meat servings per week				0.52
None	268 (8.1)	264 (8.1)	4 (11.8)	
1 serving	2577 (77.9)	2550 (77.9)	27 (79.4)	
More than 2	462 (14.0)	459 (14.0)	3 (8.8)	
Type of oil/fat used for meal				**0.01**
Olive oil	388 (11.7)	378 (11.5)	10 (29.4)	
Animal fat	14 (0.4)	14 (0.4)	0 (0.0)	
Butter	9 (0.3)	9 (0.3)	0 (0.0)	
Margarine	1 (0.0)	0 (0.0)	1 (2.9)	
Vegetable oil	2039 (61.7)	2020 (61.7)	19 (55.9)	
Vegetable oil and olive oil	554 (16.8)	550 (16.8)	4 (11.8)	
Vegetable oil and animal fat	199 (6.0)	199 (6.1)	0 (0.0)	
Vegetable and butter	60 (1.8)	60 (1.8)	0 (0.0)	
Olive and butter	16 (0.5)	16 (0.5)	0 (0.0)	
Time usually spent sitting or reclining/day in hours *Median (IQR)*	5.0 (3.0–8.0)	5.0 (3.0–8.0)	5.0 (3.0–6.0)	0.82
Physical activity/week				0.23
None	1518 (45.9)	1503 (46.0)	15 (44.1)	
Irregularly	810 (24.5)	805 (24.6)	5 (14.7)	
Regularly	976 (29.5)	962 (29.4)	14 (41.2)	
Time usually spent doing physical activity in h/week *Median (IQR)*	28.0 (14.0–42.0)	28.0 (14.0–42.0)	42.0 (14.0–50.7)	0.459
Type of physical activity performed				0.954
Did not specify	18 (1)	18 (1)	0 (0)	
Housework	1266 (70.9)	1253(71.0)	13 (68.4)	
Leisure	54 (3)	53 (3)	1 (5.3)	
Occupational	206 (11.5)	204 (11.6)	2 (10.5)	
Occupational and housework	197 (11)	195 (11)	2 (10.5)	
Housework and leisure	43 (2.4)	42 (2.4)	1 (5.3)	

*p*-values were calculated using either the chi-square test or the independent *t*-test, where *p* < 0.05 is significant. IQR: interquartile range.

**Table 6:** Multivariable logistic regression analysis showing predictors of positive screening.

	Odds Ratio	95% Confidence Interval	*p*-Value
History of breastfeeding	0.522	(0.147–1.848)	0.313
Age at menarche	1.008	(0.791–1.284)	0.947
First mammogram ever	1.695	(0.487–5.898)	0.407
Age at menopause	0.974	(0.906–1.048)	0.489
Result of clinical examination	21.257	(7.913–57.108)	<0.0001
History of contraceptive pill use	1.665	(0.513–5.399)	0.396
Length of lifetime use of contraceptive pills	1.003	(0.997–1.009)	0.268

*p* < 0.05 is significant.

## Data Availability

The datasets from the current study are available from the corresponding author upon reasonable request.

## Supplementary Materials

The following supporting information can be downloaded at: https://www.mdpi.com/article/10.3390/cancers17213403/s1, Supplementary Document S1: Breast cancer risk assessment tool.
