# Supplementary material for: Characteristics of Participants and Findings of the National Breast Cancer Early Detection Program in Saudi Arabia
Source: Cancers (Basel). 2025 Oct 22;17(21):3403. doi: 10.3390/cancers17213403 (PMC12610800; doi:10.3390/cancers17213403)
Supplement: Supplementary file 1 [file cancers-17-03403-s001.zip › Supplementary Document S1.pdf]

NATIONAL BREAST CANCER EARLY DETECTION PROGRAM

Breast Cancer Risk Assessment Tool

01) ID Number: ☐ ☐ ☐ ☐ ☐ ☐ ☐ ☐ ☐ ☐

02) Date of Examination: (/ /).

03) Age: **04) Date of Birth:** (/ /).

05) Weight (Kg) : **06) Height (meters) :**

07) What is the Highest Level of Education you Have Completed?
a. literacy b. Primary school completed c. Secondary school completed
d. High school completed e. College/ university complete f. post-graduate degree

08) Do you have diabetes ? a: Yes b: No**:**
08-1) at what age started**::**
08-2) Do you take insulin: a: Yes b: No
08-3) Diabetes: Type I Type II I don't know:

09) Is this your first mammogram ever? a.Yes b. No
If yes,
09-1How many mammograms have you had before?
09-2What was the date of your last mammogram? (/ /).

10) Who referred you/ advised you to come for a mammogram?
a. Doctor b. Self-referred c. Family member: Specify: d. Friend
e. health educator f. Other

11) Menstrual history:
11-1 age at menarche:
11-2 When did you have your last menstrual period: (/ /)
11-3 Have you gone through menopause? Yes No (if yes):
11-4 Age at Menopause::

12) Are you currently: Married Single Divorced Separated Widowed

13) If married or previously married, please answer the questions that follow, otherwise proceed to question#15
13-1 Have you been pregnant: Yes No (if No, please proceed to Question #14)
13-2 How many times have you been pregnant?
13-3 Age at first full-term pregnancy (FTP) / live-birth:
13-4 Total number of FTPs:
13-5 Total number of live births:

14) have you breastfed your children: Yes No
If Yes:
what was the total time you have breastfed them all?

15) Have you ever used any oral contraceptives: Yes No

If Yes:

15-1 How long have you been using / used the pills (lifetime-use):

15-2 If stopped using the pills, how many years since stopping:

16) Have you ever used hormonal Replacement Therapy (HRT): Yes No

If Yes:

16-1 How long have you been using the HRT pills (lifetime use)

16-2 If stopped, how many years since stopping**:**

17) Have you ever had a breast biopsy: Yes No

17-1 If yes: how many:

17-2 Is any of these breast biopsies had abnormal findings? Yes No

18) Were you ever diagnosed with breast cancer? Yes No which age:

19) Were you ever diagnosed with ovarian cancer: Yes No which age:

20) Were you ever exposed to mantle radiotherapy between 10-30 years of age: Yes No

21) Has any of your family members been diagnosed with breast cancer or ovarian cancer: Yes No

**a. Which first degree relative had?**		**What was their age at time of diagnosis?**	
**breast cancer**	**Ovarian cancer**	**breast cancer**	**Ovarian cancer**
Mother	Mother		
Sister	Sister		
Daughter	Daughter		
Father, brother			

**b. Which second degree relative had?**		**What was their age at time of diagnosis?**	
**Breast cancer**	**Ovarian cancer**	**Breast cancer**	**Ovarian cancer**
Grandmother/Grandfather	Grandmother		
Aunt 1	Aunt 1		
Aunt 2	Aunt 2		
Niece (brother's daughter)	Niece (brother's daughter)		
Niece (sister 's daughter)	Niece (sister 's daughter)		

22) Diet:

a. How many servings of fruits &vegetables **per day/per week**: None One serving 2-4 servings 5 or more

b. how many serving of red meat and meat products consumed per **day/per wee**k:

None 1-2serving 2 serving or more

c. What type of oil/fat is most often used for meal preparation in your household?

Vegetable oil olive oil Animal fat Butter Margarine None

23) Physical Activity:

a. On a typical day, how much time do you usually spend sitting or reclining?

b. In a typical week, do you perform any type of physical activity (example: walking or other types of exercise)?

Regularly irregularly No

c. If yes, how much time per week do you spend doing these activity/ activities?

d. Physical activities are mostly:

Occupational Housework leisure

24) Findings of Clinical Examination: negative Positive If Positive**:**

a. Breast change: Breast lump/ Breast thickening / Asymmetry / pain / size change / shape change

b. Nipple: bloody/ serious discharge/ inversion / pain

c. Skin: rash / scaly / change in color

d. Lymph node enlargement**:** Yes No

25) What is the reason for your referral to mammogram:

25-1 Being 40 years & above

25-2 Having risk factor for breast cancer

25-3 Having breast changes/ symptoms

Name:

Signature:

Date:
